# Enhancing the Flexural Strength of AlN with an Additional Cross-Linking Mechanism in the Aqueous Isobam Gelling System

**DOI:** 10.3390/ma17143410

**Published:** 2024-07-10

**Authors:** Yixuan He, Xiaohong Wang, Ning Ding, Hai Jiang, Wenzhong Lu

**Affiliations:** 1 School of Integrated Circuits, Huazhong University of Science and Technology, Wuhan 430074, China; m202272764@hust.edu.cn (Y.H.); 15071460872@163.com (N.D.); 2 Wenzhou Advanced Manufacturing Institute, Huazhong University of Science and Technology, Wenzhou 325035, China; jianghai@nbu.edu.cn (H.J.); lwz@hust.edu.cn (W.L.); 3 Department of Microelectronic Science and Engineering, Ningbo University, Ningbo 315211, China

**Keywords:** gelcasting, Isobam, aluminum nitride, flexural strength, cross-linking

## Abstract

Isobam is widely used for fabricating ceramics through spontaneous gelation and has attracted considerable interest. However, the disadvantage of the Isobam system is the low gelation strength. The effects of suitable additives and the mechanism by which they effectively enhance the green body strength and the rheological behavior of an aluminum nitride (AlN) slurry with 50 vol% solid loading were investigated using polyethyleneimine (PEI), hydantoin epoxy resin, and trimethylolpropane triglycidyl ether (TMPGE). Results showed that the additives acted as both dispersants and cross-linkers in the AlN suspension using the Isobam gelling system. The flexural strength of the AlN green body increased by 42%, 204%, and 268% with the addition of 1 wt% PEI, 1 wt% hydantoin epoxy resin, and 0.5 wt% TMPGE, respectively. After sintering at 1700 °C, the AlN ceramic with 0.5 wt% TMPGE had flexural strength and thermal conductivity of 235 MPa and 166.44 W/(m·K), respectively, showing superior performance to the ceramics without additives.

## 1. Introduction

Gelcasting is a colloidal forming technique for ceramics and has many attractive merits. A typical gelcasting process involves mixing ceramic powders with a monomer, cross-linker, initiator, and catalyst in solvent to form a low-viscosity suspension that can be easily cast in molds and form solidified three-dimensional macromolecule networks that trap ceramic particles. Therefore, homogeneous green bodies with various shapes are obtained [1]. Gelcasting allows for the formation of dense, homogeneous, complex, and highly accurate shapes with high green body strength, thus lowering requirements for molds and other equipment [1,2,3,4]. The first gelcasting system proposed by Oak Ridge National Laboratory uses acrylamide (AM) as the monomer and methylene bisacrylamide as the cross-linker. The system produces gel cross-linking networks with entrapped powders through free radical polymerization after the addition of initiators and catalysts [5]. However, AM is neurotoxic [6], and thus alternative monomers with low or no toxicity have been explored [7,8,9]. Some cross-linkable binder systems based on nucleophilic addition reactions have been developed, such as epoxy resin and 3,3′-diaminodipropylamine (DPTA) [10,11]. Some natural gels have also been developed, such as agarose [12] and protein [13], because they are environmentally friendly.

Yang et al. [14] discovered that 50 vol% alumina slurries can spontaneously solidify at room temperature and form elastic green bodies with 0.3 wt% copolymer composed of isobutylene and maleic anhydride (Isobam104; M.W. = 55,000–65,000; water-soluble), in which Isobam104 acted as both gelling additive and dispersant because of its functional groups: –CONH_2_, –COO–, and –CO–O–CO– [15]. Only a small quantity of Isobam104 (lower than 1 wt%) can produce a high solid content suspension with low viscosity, and the slurry can spontaneously solidify within a specific period [16]. These features are conducive to gelcasting, debinding, and production of dense ceramics. Moreover, Isobam is nontoxic and water soluble [15]. Compared with other gel processes, Isobam-induced spontaneous coagulation produces gel networks that are conducive to water transport. In addition, internal stress generated by the green body during drying is small, and the deformation and cracking of the green body are eased. Owing to the unique characteristics of Isobam, Isobam spontaneous coagulation casting has attracted much attention. Isobam is widely used in the preparation of various non-oxide or oxide ceramics, such as SiC [17], Si_3_N_4_ [18], Al_2_O_3_ [19], YAG [20], Y_2_O_3_ [21], and AlON [22]. Its gelation mechanisms have been extensively explored [23,24,25,26], including electrostatic interactions, hydrophobic coagulation, physical entanglement, and hydrogen bonding among its main chains adsorbed on adjacent ceramic particles. In contrast to a traditional gelation system, the ceramic particles also participate in generating gel networks for the Isobam gel system.

However, the green bodies of Isobam gelling systems have low strength because of the weak connections and absence of chemical bonds in spontaneous solidification. Low green body strength is unfavorable to the formation of large and complex-shaped products and can cause cracks during transport, reducing performance and damaging the resulting ceramics. Moreover, the dispersion ability of Isobam is insufficient [27], which leads the viscosity to increase greatly for the suspension with high solid loading. Wang et al. [28] used 0.15 vol% polyethylene imine (PEI) as a cross-linker for 50 vol% mullite slurry with 3 vol% Isobam110. After solidifying at 60 °C, a green body with high flexural strength (12.5 ± 0.6 MPa) was obtained. Sun et al. [29] improved the flexural strength of an alumina green body by introducing ethylene glycol diglycidyl ether. Su et al. [30] combined Isobam with a traditional AM gel-casting process to prepare high-strength B_4_C ceramics. Zhang et al. [31] found that 0.2 wt% tetramethyl ammoniumhydroxid pentahydrate (TMAH) formed –N(CH_3_)_4_ or –COON(CH_3_)_4_ groups, promoted the hydrophobicity of Isobam, and improved the strength of alumina. Ding et al. [26] enhanced hydrogen bonding by adding 1 wt% ammonium persulfate to aluminum nitride (AlN) slurry, and the flexural strength of the AlN green body improved by 48%, reaching 2.5 MPa. However, the increase was limited because the hydrogen bonds were weak.

As an insulation dielectric with high thermal conductivity, AlN is widely employed in the field of electronics [32]. Apart from the thermal conductivity of materials, heat dissipation can be improved by modifying structures [33]. Therefore, Isobam gelcasting can satisfy the increasing demand for AlN ceramics with various shapes. However, the surface charge of commercial AlN with water-resistant treatment is approximately electroneutral [26], and the weak electrostatic interaction between the particles and Isobam results in a low-strength green body.

To offset the limitations of the Isobam gelation system, some polymers with amine or epoxy groups were added to AlN slurry in this study based on the following considerations. First, these polymers can provide sufficient steric or electrostatic stabilization, serving as dispersants and reducing the viscosity of slurries. Second, polymers adsorbed on the AlN particle surfaces acted as cross-linkers through polymerization with the functional groups of Isobam, which formed extra gelation networks and enhanced the strength of the AlN green body. Furthermore, the additives showed different aqueous solubility. According to the gelation mechanism of Isobam, the hydrophobic additive enhanced the hydrophobic binding and hydrogen bonding. In this way, the cross-linking of the Isobam gelation system was enhanced by the polymer dispersant. Therefore, the purpose of this study was to investigate the gelation and rheological properties of AlN suspensions with the addition of PEI, hydantoin epoxy resin, and trimethylolpropane triglycidyl ether (TMPGE). The effects of the three additives on the strength of the AlN green bodies and sintered ceramics were explored, and the enhanced gelation mechanism of AlN was investigated through infrared spectroscopy.

## 2. Materials and Methods

Commercial AlN powder (grade: TFZ-A02P, with water-resistant treatment, Toyo Aluminum, Osaka, Japan) and sintering additions of Y_2_O_3_ (99.99%) and YF_3_ (99.9%) were mixed by ball-milling and dried (referred to as AlN-S powder). Isobam104 (99%; Kuraray, Tokyo, Japan) was applied as the gelling agent. PEI (99%, Aladdin, Shanghai, China), hydantoin epoxy resin (MHR-070, Technical Grade, Bonuo Biotechnology, Nanjing, China), and TMPGE (99%, Huaxiangkejie Biotechnology, Wuhan, China) were introduced as additives to build new gel networks with Isobam104. To examine the gel behavior of the Isobam with the various additives, Isobam104, different additives, and deionized water were first blended in beakers by magnetic stirring for 15 min. Then, the solutions were placed in an oven at different temperatures for gelation. The AlN ceramics were prepared from the AlN suspension with a solid loading of 50 vol%. AlN-S powder, deionized water, 0.5 wt% Isobam104, 1 wt% PAA–NH_4_ (40% in water, Maya Reagent, Wulumuqi, China), and *x* wt% (*x* = 0–1.5) additive (selected from PEI, hydantoin epoxy resin, and TMPGE) were mixed at 200 revolutions per minute for 2 h through ball-milling for the preparation of 50 vol% AlN suspensions. After vacuum degassing for 10 min, the AlN suspensions were poured into molds. The gelled wet green bodies were dried at 50 °C and molded out. After the organics were burned at 550 °C, the AlN bodies were sintered at 1700 °C for 3 h in a graphite furnace with flowing N_2_.

The viscosity of the mixed Isobam104 and additive solution was measured using a rotary viscometer (SNB-2, Shanghai Institute of Geoscience Instruments, Shanghai, China) at 60 rad/min. Differential scanning calorimetry was carried out for the solution of Isobam and additives using a thermogravimetric analyzer (TGA 8000, PerkinElmer, Waltham, MA, USA) with a heating rate of 8 °C/min. The infrared spectrum was measured using an FT-IR spectrometer (Nicolet iS50R, Bruker, Karlsruhe, Germany). The rheological behavior of the AlN slurries was analyzed with a viscometer (DV2T, Brookfield, MA, USA). The flexural strength of the rectangular AlN green bodies and ceramics was measured using a universal tester (Z020, Zwick Roell, Ulm, Germany) with a span of 30 mm. The fractured surfaces of the AlN ceramics were observed through scanning electron microscopy (EM-30 PLUS, Coxem, Daejeon, Republic of Korea). The thermal conductivity of the AlN ceramics was determined using laser thermal conductivity (LFA 467 HyperFlash, Netzsch, Selb, Germany).

## 3. Results and Discussion

### 3.1. Effect of Additives on the Gelation Behavior and the Enhanced Gelation Mechanism

**Figure 1:**
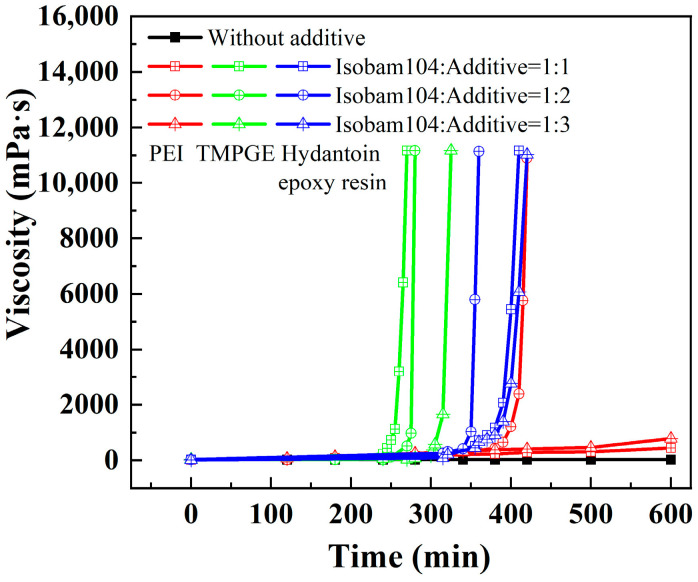
Viscosity versus time for the Isobam104 solution with PEI, hydantoin epoxy resin, and TMPGE.

Gelation behavior was observed in prepared Isobam–additive (PEI, hydantoin epoxy resin, and TMPGE) aqueous solutions. Figure 1 illustrates the viscosity of the aqueous solutions with different ratios of Isobam to additive (PEI, hydantoin epoxy resin, and TMPGE) at 50 °C and shear rate of 60 rad/min. The viscosity of solutions containing only Isobam104 is as low as 28 mPa·s and invariable, indicating the absence of gelling behavior. The reason is that the main interactions due to the hydrogen bonds in the Isobam solution without ceramic powder are extremely weak [14,34]. After the addition of PEI, hydantoin epoxy resin, or TMPGE, the viscosity of the mixed aqueous solution changes and increases rapidly: these effects indicate that the additives cross-linked with Isobam104 and formed gel networks. As shown in Figure 1, the gelation behavior varies with the kind and content of the additive. Gelation time in the Isobam–TMPGE system is the shortest when the content of the additive is the same. With increasing additive content, the gelation time decreases first and then increases. For example, when the ratio of Isobam104 to hydantoin epoxy resin in the solution is 1:1, the gelation time of the solution is approximately 400 min. When the ratio reaches 1:2, the gelation rate of the solution is rapid, and the gelation time is approximately 350 min. This is because the additive reacts with Isobam104 [29], and the possibility of reaction between epoxy resin and gelling agents is higher with a high additive concentration. However, when the content of hydantoin epoxy resin increases further, all reaction sites on one Isobam104 chain will react with epoxy resin. Anhydride groups remaining on the chain of Isobam104 and their interactions are reduced. Therefore, the gelation rate of the mixed solution decreases. This result is similar to the results of Wu et al. [35]. Excessive amounts of hydantoin epoxy resin hinder the formation of the gel networks, and the optimal amount of hydantoin epoxy resin is twice that of Isobam104. Similarly, the preferred ratio of Isobam104 to PEI is 1:2. In the Isobam104–TMPGE system, the gelation time increases when the ratio of Isobam104 to TMPGE decreases from 1:1 to 1:3. This implies that the required amount of TMPGE to react with Isobam104 is low.

**Figure 2:**
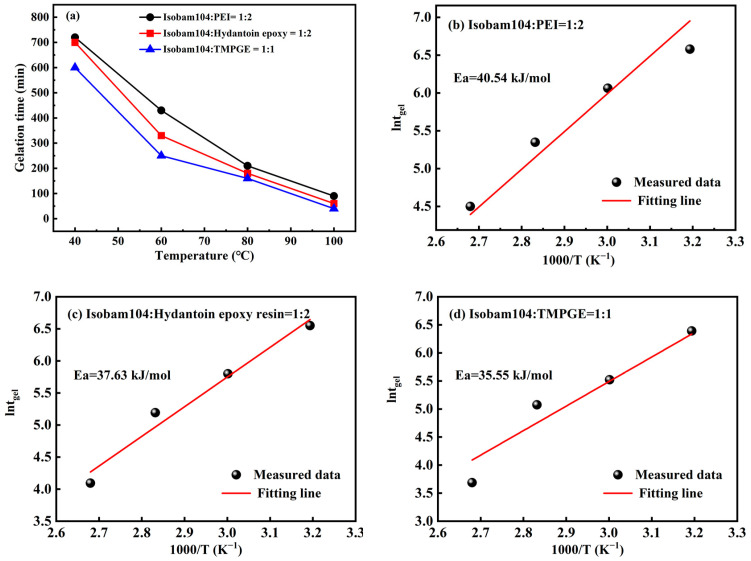
(**a**) Gelation time with temperature for the Isobam104 aqueous solutions with different additives. The Arrhenius plots obtained for Isobam104 solution with (**b**) PEI, (**c**) hydantoin epoxy resin, and (**d**) TMPGE.

To offset the influence of stirring on gelation, gelation time (*t_gel_*) was recorded when the liquid stopped flowing rather than being obtained from the viscosity curve. Figure 2a shows the influence of temperature on the gelation time of the Isobam104 aqueous solution with PEI, hydantoin epoxy, or TMPGE. The gelation time decreases with increasing gelling temperature. Compared with PEI and hydantoin epoxy resin, TMPGE has a lower gelation time. When the temperature exceeds 80 °C, the difference in gelation time for the three kinds of mixed solutions is small. Although the gelation time is shortened at high temperatures, the accelerated cross-linking reaction causes moisture in the green bodies to evaporate rapidly and leads to cracking, which should be avoided. Gelation time (*t_gel_*) is inversely proportional to the reaction rate *k*, which is described by the Arrhenius equation [36]:

(1) $$t_{gel}\propto \frac{1}{k}=Aexp\left(\frac{E_{a}}{RT}\right)$$

where *Ea* is the activation energy, *T* is the temperature in Kelvin, R is the gas constant, and A is a constant. The plot of $lnt_{gel}$ versus 1000/*T* and the calculated *Ea* from the slope of the fitting line for Isobam104 with different additives are shown in Figure 2b–d. The *Ea* values calculated from Figure 2b–d are 40.54, 37.63 and 35.55 kJ/mol for the mixed aqueous solutions with Isobam104–PEI, Isobam104–hydantoin epoxy, and Isobam104–TMPGE ratios of 1:2, 1:2, and 1:1, respectively. The *Ea* value of Isobam–TMPGE is the lowest, indicating that the reaction rate between TMPGE and Isobam104 is the fastest under the same conditions. As such, the influence of TMPGE on the gelation behavior of Isobam104 solution is the strongest, as shown in Figure 1. Generally, more organic additive results in more pores after burnout, which deteriorate the performance of ceramics after sintering. Given the activation energy and addition content, TMPGE is the preferred additive.

**Figure 3:**
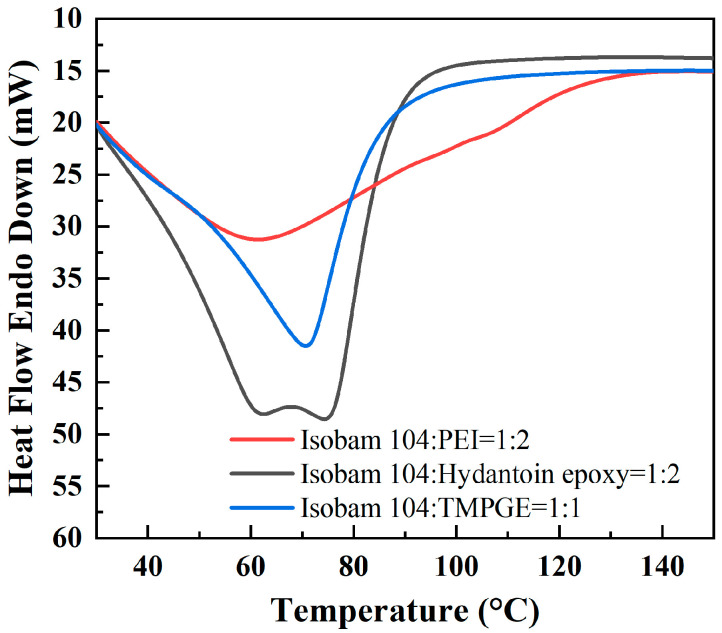
Heat flow–temperature curves for Isobam104 aqueous solutions with different additives.

Figure 3 shows the heat flow–temperature curves for the Isobam104 solution with different additives. The exothermic peaks indicate that there are curing reactions. As illustrated in Figure 3, the initial curing temperature is room temperature.

**Figure 4:**
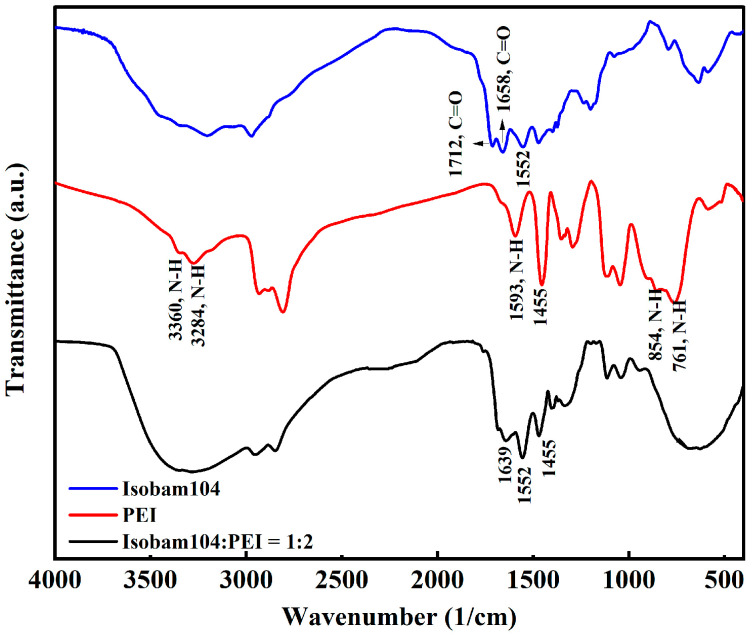
Infrared spectra of Isobam104, PEI, and Isobam–PEI gel (1:2).

As shown in Figure 1 and Figure 3, the additives with different structures can form gel with Isobam104. The mechanism was determined by investigating the infrared spectrum. The infrared spectra of Isobam104, PEI, and xerogel with Isobam and PEI in a 1:2 ratio are shown in Figure 4. Compared with the infrared spectrum of Isobam, the infrared spectrum of the Isobam–PEI gel showed changes. First, the 1658 cm^−1^ peak associated with the C=O stretching vibration from the –CONH_2_ of Isobam shifts downward to 1639 cm^−1^ because of the formation of hydrogen bonds in –CONH_2_ [37]. Second, the peak at 1712 cm^−1^ corresponding to the C=O stretching vibration from –CO–O–CO– of Isobam completely disappears. The disappearance at 1712 cm^−1^ indicates the hydrolysis of anhydride groups in Isobam104 and subsequent production of a –COOH group [26]. However, no peak at 1700–1725 cm^−1^ corresponding to the C=O stretching from the –COOH group appears. Thus, the –COOH group produced by the hydrolysis of the anhydride group of Isobam104 may have reacted with PEI. The appearance of peaks around 1639 cm^−1^ corresponding to the C=O stretching vibration from –CONH– indicated that the –COOH group polymerizes with the amino group of PEI. As illustrated in Figure 3, the peak at 1593 cm^−1^ (N–H bending vibration of primary amines), peaks at 910–655 cm^–1^ (N–H wag of primary and secondary amines), and a pair of peaks at 3000–3300 cm^−1^ (N–H stretching modes of primary amines) [38] are observed in the FTIR spectrum of PEI. However, the 1593 cm^−1^ and 910–655 cm^−1^ peaks associated with the N–H vibration of primary amines disappear in the Isobam–PEI gel. In addition, only one broadened band appears in the 3400–3250 cm^−1^ range. The results indicate that the primary amines of PEI are involved in the polymerization, as shown in Supplementary Figure S1. Thus, a gel network forms through hydrogen bond interaction and chemical cross-linking [28].

**Figure 5:**
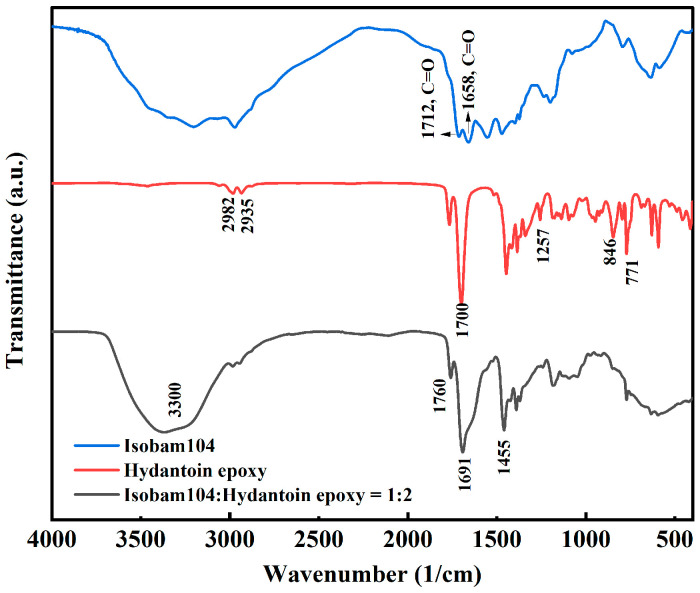
Infrared spectra of Isobam104, hydantoin epoxy resin, and gel of Isobam104–hydantoin epoxy resin with a ratio of 1:2.

Figure 5 shows the infrared spectra of Isobam104, hydantoin epoxy resin, and Isobam–hydantoin epoxy resin gel (1:2). As shown in Figure 5, the peak at 1712 cm^−1^ of Isobam disappears due to hydrolysis. This effect was also observed in the Isobam–PEI system. Compared with the infrared spectrum of hydantoin epoxy resin, the characteristic absorption bands of epoxy groups at 1280–1230 cm^−1^ (symmetric ring breathing vibration), 950–810 cm^−1^ (asymmetric C–O–C stretch), and 880–750 cm^−1^ (C–O–C stretch) [39] disappear in the gel of the Isobam-hydantoin epoxy resin system. A new peak at 1760 cm^−1^ corresponding to the C=O stretching vibration in the ester is found. Therefore, in situ polymerization occurs between the epoxy group in the hydantoin epoxy resin and the amide or carboxylic acid group generated by the hydrolysis of anhydride in Isobam104 through ring opening, resulting in the formation of esters. As presented in Figure 5, a wide peak at approximately 3200 cm^−1^ was detected in the gel of Isobam104 and hydantoin epoxy resin, which is related to the vibration of the O–H bond and N–H stretch in secondary amines [38]. It indicates that the epoxy group reacts with the carboxylic acid hydrolyzed by Isobam104. The reaction produces esters and hydroxyl groups. Based on the results of the infrared spectrum, the reaction mechanism between hydantoin epoxy resin and Isobam104 is schematically illustrated in Supplementary Figure S2. The carboxylic acid produced by the hydrolysis of Isobam104 reacts with the epoxy group in the hydantoin epoxy resin to form esters and hydroxyl groups, which can further react with carboxylic acid to form esters. Therefore, Isobam molecular chains can be connected through hydantoin epoxy resin to form an additional three-dimensional gel network.

**Figure 6:**
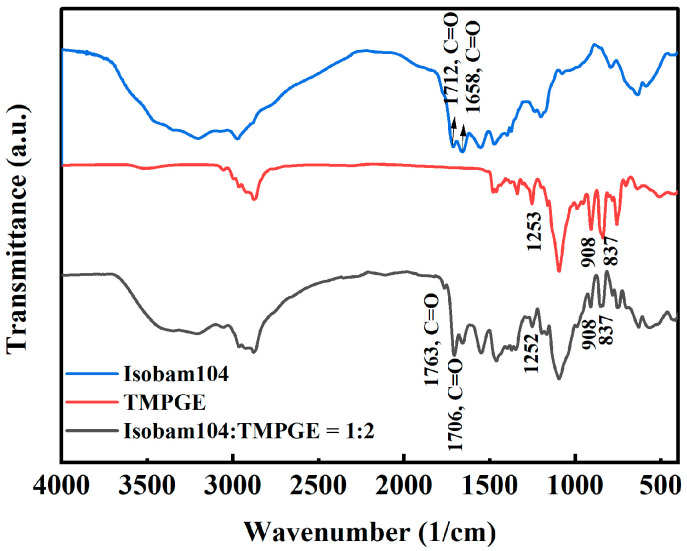
Infrared spectra of Isobam104, TMPGE, and gel with Isobam104–TMPGE with a ratio of 1:2.

The infrared spectra of Isobam, TMPGE, and the gel with Isobam–TMPGE ratio of 1:2 are illustrated in Figure 6. The peak at 1712 cm^−1^ disappears and a new peak at 1763 cm^−1^ appears in the Isobam–TMPGE gel, similar to the results shown in Figure 6. Given that TMPGE contains epoxy groups, the gelation mechanism is the same as that in the hydantoin epoxy resin. However, peaks at 1253, 908, and 837 cm^−1^ are detected in the Isobam–TMPGE system. These peaks correspond to symmetric ring breathing vibration and asymmetric and symmetric C–O–C stretching vibration from epoxy groups [39]. As shown in Figure 1, the optimal ratio between TMPGE and Isobam is 1:1. Excessive TMPGE leads to residual epoxy groups in the gel, with an Isobam–TMPGE ratio of 1:2 in Figure 6. Compared with hydantoin epoxy resin containing two epoxy groups in molecules, a TMPGE molecule contains three epoxy groups, and thus the optimized additive amount is low and the excessive TMPGE exists alone.

### 3.2. Effect of Additives on the Properties of AlN Slurry, Green Body, and Ceramic

**Figure 7:**
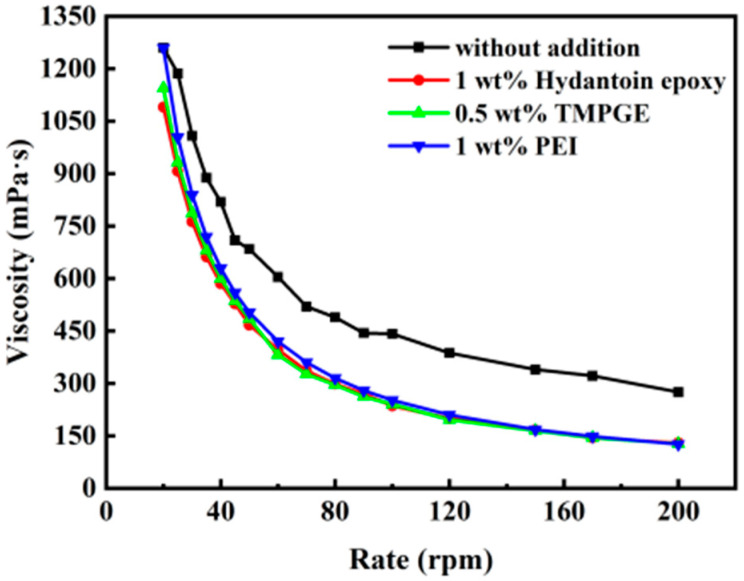
Rheological properties of AlN slurries with different additives.

Figure 7 shows the rheology of 50 vol% AlN–0.5 wt% Isobam104–1 wt% PAA–NH_4_ slurries with 1 wt% PEI, 1 wt% hydantoin epoxy resin, and 0.5 wt% TMPGE. The viscosity of all suspensions decreases with increasing shear rate and the slurries exhibit typical shear-thinning behavior. Moreover, the viscosity at 100 rpm decreases significantly from 442 mPa·s for the AlN slurry without additive to 252, 236, and 240 mPa·s with the addition of PEI, hydantoin epoxy, and TMPGE, respectively. Owing to the enhancement in steric and electrostatic stabilization after the addition of additives with high molecular weight and long chains, the viscosity of the slurry decreases, which is favorable for gel casting. Therefore, the additive (PEI, hydantoin epoxy, or TMPGE) acts not only as a cross-linker but also as a dispersant in the AlN slurry.

**Figure 8:**
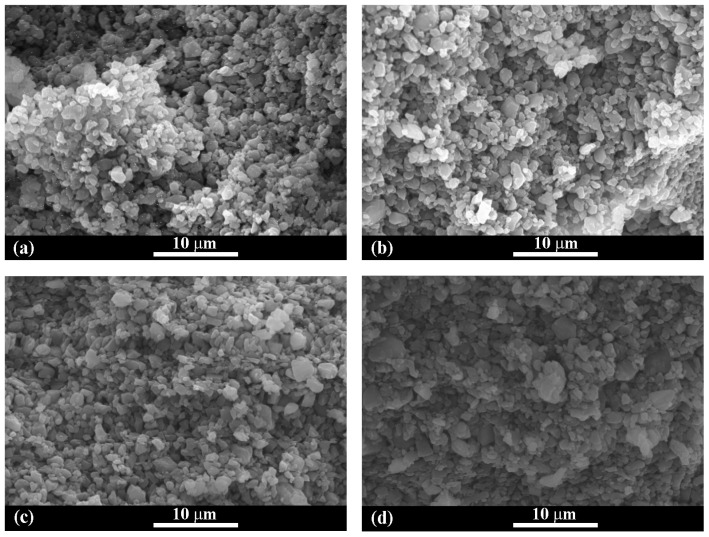
SEM images of the fractured surface of the dried AlN green bodies (**a**) without additives, (**b**) with 1 wt% PEI, (**c**) with 1 wt% hydantoin epoxy resin, and (**d**) with 0.5 wt% TMPGE.

After casting in molds, all of the AlN slurries transform spontaneously to solid wet body at room temperature. The results indicated that AlN particles also participate in the formation of gelling networks. However, the gelation time of the AlN slurries without additives is as long as 48 h at room temperature. The gelation time is shortened to 36 h, 24 h, and 22 h for AlN slurries with 1 wt% PEI, 1 wt% hydantoin epoxy resin, and 0.5 wt% TMPGE, respectively. The microstructures of the fractured surfaces of the dried green bodies with and without the optimal content of additives were observed by SEM. As shown in Figure 8a,b, some AlN particles tightly connect and aggregate together, while other particles are looser. With the addition of hydantoin epoxy resin or TMPGE, the morphologies in Figure 8c,d demonstrate a homogeneous microstructure and AlN particles are packed more tightly on the whole, which is favorable for improving the flexural strength.

**Figure 9:**
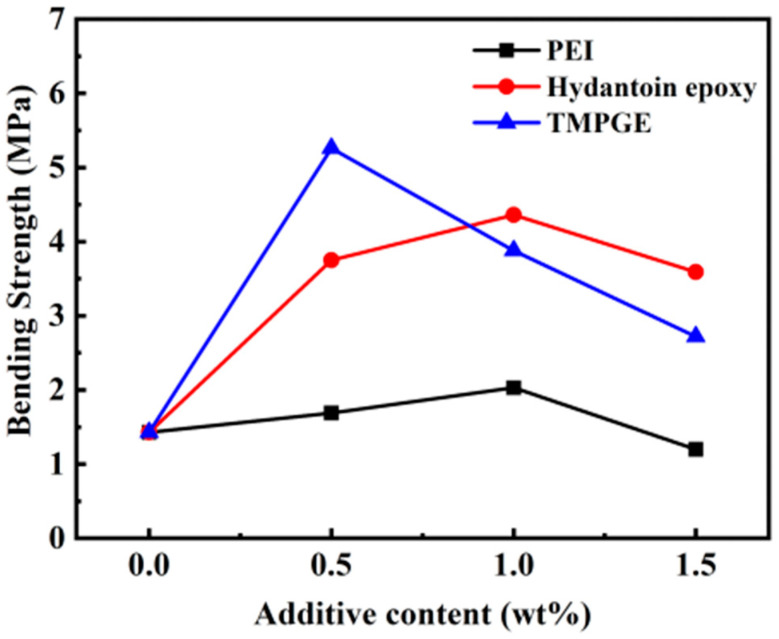
Flexural strength of the AlN green bodies obtained for the slurries with different amounts of additives.

Figure 9 illustrates the flexural strength of the green bodies prepared from 50 vol% AlN slurries with different additives. The flexural strength of the AlN green bodies first increases and then decreases with the increasing additive content. In general, flexural strength is improved by the compact three-dimensional gel network and high cross-linking density. Maximum flexural strengths of 2.03, 4.36, and 5.26 MPa were obtained at 1.0 wt% PEI, 1.0 wt% hydantoin epoxy, and 0.5 wt% TMPGE, respectively, consistent with the optimal ratio from Section 3.1. Compared with the AlN green body without an additive, the AlN green body exhibits considerable improvement in flexural strength for the chemical gel network formed between Isobam and an additive, especially with the addition of 1 wt% hydantoin epoxy and 0.5 wt% TMPGE, which results in 204% and 268% increases, respectively. As shown in Figure 3, the temperature range of the exothermic peak is similar in hydantoin epoxy resin and TMPGE, and the range of PEI is wide. This indicates that the curing reaction ends later for PEI under the same conditions. Moreover, the molecular chain of PEI is long. Thus, compared with PEI, the cross-linked network formed is sufficiently strong for hydantoin epoxy resin and TMPGE. However, excess additives decrease the mechanical strength. As shown in Figure 1, the gelation time of the mixed solution increases when the ratios of PEI, hydantoin epoxy, and TMPGE with Isobam exceed 2:1, 2:1, and 1:1, respectively. AlN particles in the suspension with high solid loading would deposit in the prolonged idle time. Thus, the long gelation time results in a deterioration in the homogeneity of the green body [29], which is consistent with the microstructure shown in Figure 9. Meanwhile, excess additives in gel (Figure 6) would produce residual stress in the green body. Thus, the inhomogeneity and residual stress reduce the flexural strength of the green bodies when the additive content increases further.

**Figure 10:**
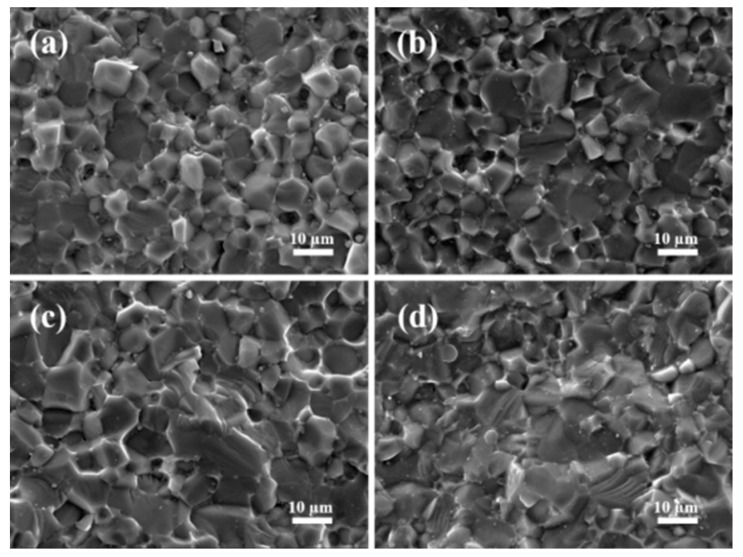
SEM images of the fractured surface for AlN ceramics: (**a**) without additives; (**b**) with 1 wt% PEI; (**c**) with 1 wt% Hydantoin epoxy resin; (**d**) with 0.5 wt% TMPGE.

The fractured surfaces of the AlN ceramics sintered at 1700 °C prepared from the 50 vol% AlN slurries with different additives are shown in Figure 10. All of the AlN ceramics exhibit a dense and homogeneous microstructure. With the addition of the cross-linker, the grain size of AlN increases. As shown in Figure 10a, most of the AlN grains are intact and unbroken in the fractured surface of the AlN ceramic. This phenomenon, which is referred to as intergranular fracture, is caused by the cracks that mainly grew along the grain boundaries. After the addition of additives, more broken AlN grains appear (Figure 10b,c). This result indicates that the cracks grow through the grains and break the gains. Thus, transgranular fracture occurs. In addition, most transgranular fractured grains are observed in Figure 10d. More transgranular fracture areas originate from the strengthened grain boundaries, which contribute to the improvement in flexural strength [40]. The density, thermal conductivity, and flexural strength of AlN ceramics are listed in Table 1. Among the three additives, the ability to improve the performance of AlN ceramics is in the order of TMPGE > hydantoin epoxy resin > PEI. As shown in Figure S1, the cross-linking network formed in the Isobam–PEI system utilizes the reactions of amino groups with the carboxylic acid groups of Isobam, and the green body strength increases by only 42%. Thus, the cross-linked network is insufficiently strong. Therefore, density increases only a little compared with the sample without additives. When the additive is hydantoin epoxy or TMPGE with epoxy groups, the ring-opening reaction occurs to form esters and hydroxyl groups, which can further react with the –COOH of Isobam. By the polymerization between the epoxide group and major functional groups (–CONH_2_ and anhydride) of Isobam104, a strong network is generated. Compared with the water-soluble hydantoin epoxy, TMPGE has more epoxy groups and is hydrophobic, so the curing ability enhances further through the polymerization and hydrophobic association curing mechanism [41]. Also, the *E_a_* value of the TMPGE–Isobam system is the lowest and the optimal addition is half that of the others. Consequently, the AlN particles are compact and dense, and strong three-dimensional networks form in the green bodies obtained from the slurries with 0.5 wt% TMPGE. The uniform and compact AlN particles are beneficial to grain growth and homogeneous microstructure. Thus, the optimal performance was obtained for the AlN ceramic with 0.5 wt% TMPGE.

## 4. Conclusions

PEI, hydantoin epoxy resin, and TMPGE form enhanced gel networks with the amino or epoxy groups in Isobam spontaneous gel systems through in situ cross-linking reactions stemming from acylation or ring-opening reactions. The results of gelation behavior analysis indicate that the in situ polymerization process occurs at room temperature and the gelation time is shortened with increasing temperature. The activation energy of the polymerization reaction is 40.54, 37.63, and 35.55 kJ/mol for Isobam104 aqueous solution with PEI, hydantoin epoxy, and TMPGE, respectively. The optimal ratios of Isobam104 to PEI, hydantoin epoxy, and TMPGE are 1:2, 1:2, and 1:1, respectively.

These additives act not only as a cross-linker but also as a dispersant. The viscosity of the AlN suspension with 50 vol% solid loading decreased from 442 mPa·s to 236–252 mPa·s with the addition of 1 wt% PEI or hydantoin epoxy resin or 0.5 wt% TMPGE. Moreover, the flexural strength of the green body improved by 42%, 204%, and 268%, respectively. The sintered AlN ceramics have a dense and homogeneous microstructure, and the grains grow with the additives. The flexural strength and thermal conductivity of AlN ceramics improve with the additives and reach 235 MPa and 166.44 W/(m·K), respectively, with the addition of 0.5 wt% TMPGE.

## Figures and Tables

**Table 1:** Properties of AlN ceramics prepared from 50 vol% AlN slurries with different additives.

Additives	Density (g/cm^3^)	Thermal Conductivity (W·m^−1^·K^−1^)	Flexural Strength (MPa)
None	3.257	154.57	189
1 wt% PEI	3.259	154.43	194
1 wt% hydantoin epoxy resin	3.266	162.33	217
0.5 wt% TMPGE	3.274	166.44	235

## Data Availability

The data presented in this study are available on request from the corresponding author due to privacy.

## Supplementary Materials

The following supporting information can be downloaded at: https://www.mdpi.com/article/10.3390/ma17143410/s1, Figure S1: Reaction mechanism between Isobam104 and PEI; Figure S2: Schematic diagram of the reaction mechanism between Isobam104 and hydantoin epoxy resin.
