# Supplementary material for: Enhancing the Flexural Strength of AlN with an Additional Cross-Linking Mechanism in the Aqueous Isobam Gelling System
Source: Materials (Basel). 2024 Jul 10;17(14):3410. doi: 10.3390/ma17143410 (PMC11277955; doi:10.3390/ma17143410)
Supplement: Supplementary file 1 [file materials-17-03410-s001.zip › materials-3069081-supplementary.pdf]

# Supplementary Materials

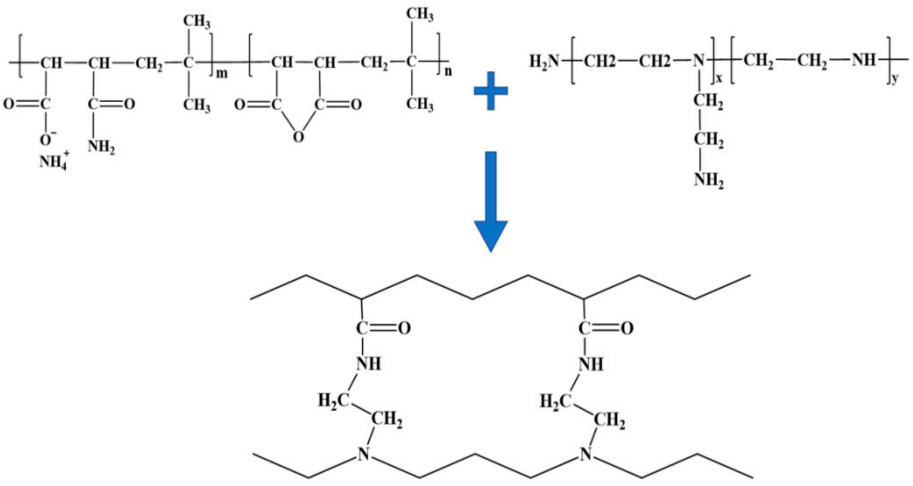


**Figure S1.** Reaction mechanism between Isobam104 and PEI.

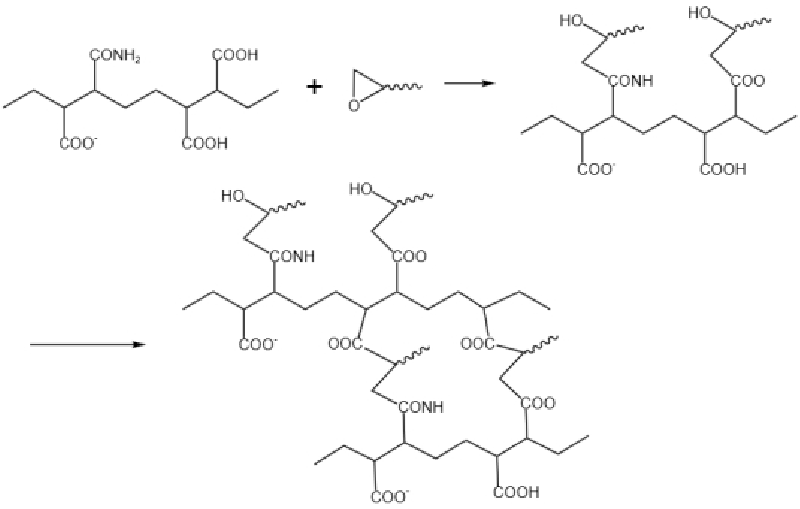


**Figure S2.** Schematic diagram of the reaction mechanism between Isobam104 and hydantoin epoxy resin.
